# Effect of Insulin on Proximal Tubules Handling of Glucose: A Systematic Review

**DOI:** 10.1155/2020/8492467

**Published:** 2020-01-10

**Authors:** Ricardo Pereira-Moreira, Elza Muscelli

**Affiliations:** Department of Internal Medicine, School of Medical Sciences, University of Campinas, Zip Code: 13083-887, Brazil

## Abstract

Renal proximal tubules reabsorb glucose from the glomerular filtrate and release it back into the circulation. Modulation of glomerular filtration and renal glucose disposal are some of the insulin actions, but little is known about a possible insulin effect on tubular glucose reabsorption. This review is aimed at synthesizing the current knowledge about insulin action on glucose handling by proximal tubules. *Method*. A systematic article selection from Medline (PubMed) and Embase between 2008 and 2019. 180 selected articles were clustered into topics (renal insulin handling, proximal tubule glucose transport, renal gluconeogenesis, and renal insulin resistance). *Summary of Results*. Insulin upregulates its renal uptake and degradation, and there is probably a renal site-specific insulin action and resistance; studies in diabetic animal models suggest that insulin increases renal SGLT2 protein content; *in vivo* human studies on glucose transport are few, and results of glucose transporter protein and mRNA contents are conflicting in human kidney biopsies; maximum renal glucose reabsorptive capacity is higher in diabetic patients than in healthy subjects; glucose stimulates SGLT1, SGLT2, and GLUT2 in renal cell cultures while insulin raises SGLT2 protein availability and activity and seems to directly inhibit the SGLT1 activity despite it activating this transporter indirectly. Besides, insulin regulates SGLT2 inhibitor bioavailability, inhibits renal gluconeogenesis, and interferes with Na^+^K^+^ATPase activity impacting on glucose transport. *Conclusion*. Available data points to an important insulin participation in renal glucose handling, including tubular glucose transport, but human studies with reproducible and comparable method are still needed.

## 1. Introduction

Diabetes global prevalence almost doubled in the last three decades. This disorder is a major cause of kidney failure, up to 44% of world end-stage renal disease, beyond ten times dialysis need and renal transplantation [1]. Kidneys are the leading organs involved in insulin clearance from the systemic circulation [2]. They contribute to endogenous glucose production through gluconeogenesis, primarily in proximal tubule (PT) cells [3] under glucose and insulin regulation [4]. Furthermore, PTs reabsorb glucose following its glomerular filtration, through the sodium-glucose linked transporters (SGLTs), mainly the SGLT2 located on the luminal surface of PT cells [5]. Consequently, renal glucose handling also depends on glucose glomerular filtration [6, 7] and on the degree of kidney damage [8].

The insulin effect has been extensively studied in renal sodium handling [9]. There is also evidence of direct [10] and indirect [11] insulin effect on glomerular filtration and modulation of renal glucose expenditure [12–14]. However, its action on renal glucose transport is still little understood.

Renal glucose uptake in diabetic patients is higher than that in healthy individuals, even when renal function is maintained [15–19]. Adaptive or pathophysiological changes in glucose transporters related to acute [20, 21] or sustained hyperglycaemia [22–26] may partially explain such difference. Nonetheless, insulin lack or resistance should be taken into consideration too.

High glucose absorption and flux, as in diabetes, may induce tubular damage via an SGLT2 dependent pathway [27, 28]. The enhanced SGLT2 activity causes mitochondrial dysfunction through a more extensive glucose flux inside PT cells [29], resulting in high oxidative stress and cellular apoptosis [30–32]. Since insulin signalling directly preserves mitochondrial metabolism and function, insulin resistance can trigger mitochondrial dysfunction and damage [33] contributing to renal injury. Reciprocally, impaired mitochondrial function reduces insulin sensitivity [33]. These findings may explain the protective effect of SGLT2 inhibition on kidneys and suggested an intrinsic relationship between renal glucose transport and insulin signalling.

Insulin has been used as a diabetes therapy since 1921 [34]. It is the principal resource to treat type 1 diabetes (T1D) as well as type 2 diabetes (T2D) patients under oral treatment failure. New therapy options include the SGLT2 inhibitors (SGLTi) that block renal glucose reabsorption and can be used as monotherapy or as add-on oral antihyperglycaemic drugs or insulin, at least in T2D patients [35]. In this way, knowing the interactions between insulin and glucose transport by PTs is important to understand not only renal diabetes impairment but also interactions among therapy drugs, mainly of insulin with SGLT2i.

This review is aimed at describing and summarizing the current understanding of the insulin effect on PTs and at discussing the main points involved in this process.

## 2. Methods

Original studies, written in English, assessing primary or secondary insulin action on glucose handling by PTs in humans, animal models, tissues, or cell cultures were eligible for inclusion. Data source, between 2008 and June 2019, from Medline (PubMed) and EMBASE, was used. Articles important to the review understanding published before 2008 and described in the references of at least one selected article were included as well.

Search terms included the following: insulin, diabetes, T1D, T2D, renal, kidney, proximal tubule or tubules, GLUT, GLUT1, GLUT2, SGLT, SGLT1, SGLT2, and Na^+^K^+^ATPase derivative terms (for example, NKA, NaKAtpase, or NKpump). We performed a triple-term search in databases with insulin, diabetes, T1D, and T2D as the first term; renal, kidney, and proximal tubule or tubules as the second; and the transport proteins as the third one. After that, an inclusive, double-term search without the second designative term was performed only in PubMed.

Two reviewers (R.P.M. and E.M.) independently evaluated the titles and abstracts and then the full text for inclusion eligibility.

Intervention studies with SGLT2i that did not evaluate insulin effect on PTs as well as those regarding glomerular function or diabetic nephropathy not related to glucose transport were excluded. Studies about renal gluconeogenesis and renal insulin resistance were included because of the possible influence of those processes on PT glucose handling.

We developed a data extraction table considering the methods and outcomes of the selected studies. One investigator extracted the data (R.P.M.) and the other reviewed it (E.M.). The extracted data included general information (title, authors, and year of publication), type of study, objectives, methodological characteristics (humans, animal models, cell cultures, renal site of evaluation, insulin intervention, isolated insulin effect, type and duration of diabetes, and insulin therapy length), and main outcomes related to the review aims.

## 3. Results

**Figure 1:**
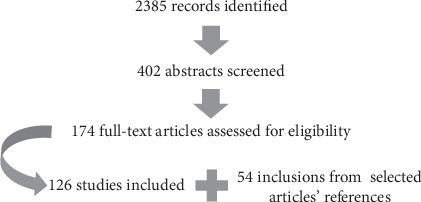
Literature flow diagram.

The articles were selected as described in Figure 1.

A total of 2385 articles were selected. After title evaluation, 1983 articles were excluded (review, not related to kidneys, to insulin action, or to glucose handling) resulting in 402 articles for abstract selection. After abstracts analysis, 228 articles were excluded with the same criteria and 174 articles were selected for a full reading. Full reading resulted in 126 selected articles from the initial search, and more 54 articles were obtained from their references. Then, a total of 180 articles were included in this review. Other 32 papers including some reviews were used to introduce and explain our aim and the result topics. The selected articles were clustered into topics and used to construct the summary of evidence described below.

### 3.1. Renal Insulin Handling

Insulin handling by the kidneys and the hormone concentration differences along the renal capillaries and tubules will be described before its action on PTs to facilitate the understanding of insulin effect at PT level and emphasize its importance.

While the liver removes around 50% of portal insulin during its first pass [36, 37], kidneys are the major organs responsible for the insulin clearance from the systemic circulation removing about 35% of total secreted insulin [2]. Most of this clearance occurs in the glomerulus impacting the hormone bioavailability in the tubular lumen and peritubular capillaries at PT level and other downstream nephron segments [2]. The majority of insulin is freely filtered in glomerular capillaries being virtually totally recovered by PT cells, predominantly across the brush border membrane (BBM), where insulin translocates through endocytic vesicles to vacuoles and then is degraded [2, 38]. Endocytosis occurs after insulin binding to the megalin-cubilin complex and, to a lesser extent, to the specific insulin receptors (IRecs) present on PT BBM. Megalin is a protein of the transmembrane complex that recovers the majority of serum proteins, including insulin. It is expressed in the PT proximal segment S1 and slightly in the intermediary S2 and distal S3 PT segments [39, 40]. Insulin increases its own uptake and degradation by inducing a rise in megalin content [41]. Less than 1% of filtered insulin undergoes transcytosis to the basolateral membrane [42], and only 1% is excreted in the urine [43–45]. The remaining nonfiltered insulin reaches the postglomerular peritubular circulation where insulin clearance takes place through specific IRecs binding mainly at PT level.

In PTs, insulin reaches its highest concentration and acts on gluconeogenesis suppression [12, 46, 47] and, possibly, on glucose transport [21, 48, 49]. Furthermore, insulin is disposed in other tubular sites where IRecs are found in high density, like the medullary thick ascending limb of Henle's loop and the distal convoluted tubules where it stimulates sodium reabsorption [50, 51].

Insulin is degraded mainly by the enzyme protein disulfide isomerase, cathepsin D, and especially the insulin-degrading enzyme (IDE). IDE is upregulated by insulin in the central nervous system, but little is known about its renal regulation [52, 53]. SNX5, a sorting nexin protein family, regulates intracellular trafficking and the expression of IRecs in PTs and upregulates IDE expression and function. The colocalization of IDE and SNX5 next to the BBM reduces insulin levels while deficiency of one or both regulators leads to increased circulating insulin levels decreasing IRec expression and inducing insulin resistance [54, 55].

### 3.2. Proximal Tubule Glucose Transport

In this section, results of experimental and clinical studies are described aiming at exploring the relationship between insulin signalling and its effect on PTs, on glucose excretion, and on renal glucose transporters, particularly in diabetes. The first topic is a brief description of renal glucose transporters, their localization, and function.

#### 3.2.1. Renal Glucose Transport Proteins

Two protein families, GLUTs and SGLTs, are in charge of the glucose transport in PT S1, S2, and S3 segments [5, 56]. GLUTs, highly expressed in kidneys, are facilitative glucose transporters present ubiquitously on cellular surfaces, composing a saturable, stereoselective, and bidirectional transport system. While GLUT1 has a high affinity for glucose, GLUT2 is a low-affinity and high-capacity transporter also mediating galactose, mannose, and fructose transport [56]. SGLTs utilize the electrochemical sodium gradient to move glucose against its concentration gradient [5]. The two types of renal SGLTs, SGLT1 and SGLT2, differ in sodium to glucose stoichiometry, sugar selectivity, sites of expression, and regulation [5, 57], even if one electrophysiological study has demonstrated similar affinities [58]. SGLT2 has higher transport capacity and is more able to adjust its glucose transport proportionally to glucose concentrations than SGLT1 [5].

In rats, GLUT1 is located in the S3 segment. It is also found in the thick limb of Henle's loop and collecting ducts [59, 60], metabolically active sites that consume large amounts of glucose as substrate [61]. GLUT2 expression has been demonstrated in the S1 segment [60, 62]. SGLT1 is found along all PT segments [59, 63], and its density in the BBM and intracellular organelles increases from S1 to S3 being higher in the outer medulla than in the cortex [63, 64]. SGLT2 is situated in the renal cortex [65], especially in the S1 and S2 segments [66, 67], and its expression is higher in the former [66]. In humans, expression of SGLT2 protein occurs in S1 and S2 whereas SGLT1 is expressed in the S3 segment. The two proteins are present only on the BBM side [57]. To our knowledge, studies regarding GLUT2 tubular localization were not performed in human but its mRNA has been demonstrated in PT cells [68–70].

Studies in knockout mice for SGLT2 or SGLT1 or SGLT2 plus SGLT1 have demonstrated that SGLT2 reabsorbs 80% to 90% glucose of the glomerular filtrate while SGLT1 reabsorbs the remaining 10-20% [71]. However, under acute [72] or chronic [73] SGLT2 inhibition or in SGLT2 knockout mice [73, 74], a compensatory increase in SGLT1-mediated glucose transport explains 40-50% of its fractional reabsorption. This is observed early, even in the first hour of SGLT2 inhibition in murine models [72]. SGLT1 vicariance justifies the maintenance of until 50% of the normal fractional glucose reabsorption during selective SGLT2 inhibition in humans [75–77]. Besides, in rats, a higher SGLT1contribution was reported under euglycaemic or hypoglycaemic conditions than in hyperglycaemic conditions [78].

#### 3.2.2. Tubular Glucose Transporters in Animal Models of Diabetes

In this topic, studies in diabetes models involving quantitative modification of a specific glucose transporter mRNA or protein were clustered (Table 1). This kind of study does not quantify the real dynamic function of the glucose transporters and their activity variation. However, all together, they can suggest transporter impairment in diabetes.

Most of the studies for GLUT1 evaluation in these models were carried out in streptozotocin (STZ) rats. They predominantly reported higher GLUT1 protein [79–82] and corresponding mRNA [81–85] contents in the whole kidney and increased GLUT1 protein [86, 87] and mRNA [86, 88, 89] in the cortex. Nonetheless, these studies are yet controversial [22, 67, 90–93]. In STZ rats, S3 GLUT1 mRNA availability raised and returned to its normal values after one month of diabetes induction, while cortical (mainly S1 and S2 segments) GLUT1 remained at low levels until six months. Subsequent insulin treatment increased the cortical but did not change the S3 GLUT1 content [24]. On the contrary, in insulin-resistant animals, GLUT1 in the S3 segment decreased in the first 3 months of diabetes and increased in the next 3 months, when cortical GLUT2 activity enhanced [25]. So, GLUT1 seems to have a differentiated regulation depending on which tubular segment is evaluated, the insulin deficiency or resistance, and the diabetes duration.

Regarding GLUT2, the results of diabetes murine models are debatable [22, 24–26, 79, 83–85, 87, 90–100]. In addition, many studies have been carried out in STZ rats [24, 26, 79, 83, 85, 87, 90–93, 96, 98], and STZ induces diabetes through beta-cell apoptosis after being transported by GLUT2 [101]. Theoretically, the same can occur in the proximal portions of PTs where GLUT2 is coupled to SGLT2. This toxicity could change the proportions of active cells impairing the evaluation of these transporters [101, 102]. In a STZ model, the increased cortical GLUT2 mRNA availability was normalized after seven days of insulin replacement [24], but glycaemic changes could have modified the results, making their interpretation problematic.

Reports of SGLT1 protein [80, 84, 103] and mRNA [83–85, 92, 93, 98] contents in PTs of T1D murine models are also contradictory while in T2D models only mRNA expression seems to be upregulated [22, 90, 92, 98, 100, 103–106]. SGLT2 has been studied in many models of diabetes, and the results suggest increased protein [32, 67, 84, 85, 90, 94, 99, 107–111] and mRNA [22, 32, 83, 85, 100, 104, 106–109, 112] contents and activity [108] despite some controversial results [85, 93, 98, 100, 105, 113].

In summary, T1D models showed increased GLUT1 in both the whole kidney and cortex. These changes can be transitory and site-specific. GLUT2 results are still controversial. SGLT1 results were concordant only regarding the upregulation of mRNA expression in T2D models. Studies frequently reported SGLT2 contents as increased in both models, a plausible reason for the higher renal glucose uptake of diabetic patients. However, whether transporter changes are due to high glycaemic levels or reduced insulin signalling or both is still an open question.

#### 3.2.3. Tubular Glucose Transporters and Renal Glucose Handling in Diabetes: Human Studies

Renal glucose reabsorption is proportional to glycaemic increments until blood glucose levels exceed the renal threshold for glucose (RTG) when glucose starts to appear in the urine [114, 115]. As glucose concentration rises above the RTG limit (around 10mmol/L [5, 15, 16]), the increment in the rate of tubular glucose reabsorption slows down in an initial nonlinear curve termed splay [116, 117]. It is followed by a constant glucose reabsorption rate that has been studied since 1940 and defines the maximum renal glucose reabsorptive capacity (Tmax). After Tmax is reached, increments in blood glucose result in equal linear increments in glycosuria [48, 116, 117].

Tmax for glucose is 15 to 20% higher in diabetic patients (356 to 463mg/min) compared to healthy subjects (303 to 404 mg/min) [15, 16, 48, 118] despite RTG variability in the former overlapping the expected RTG of the latter [17–19, 119]. The RTG seems to be increased in patients with T2D, especially in the elderly and those with long diabetes duration and higher body mass index [19]. In these patients, supposed to be the best candidates for SGLT2 inhibition because of their high RTG, the damaged kidney structure and its reduced function may impair the expected glycosuric response. In fact, a better SGLT2i effect is observed in younger diabetic patients [28].

The few studies carried out in kidneys from T2D patients reported decreased [70] or unchanged [68] GLUT1 mRNA levels while GLUT2 mRNA was described as reduced [68] or raised [100]. In exfoliated PT cells, isolated from the urine of T2D patients and cultured in a hyperglycaemic environment, GLUT2 and SGLT2 protein and mRNA were increased compared to healthy controls [69].

In diabetic patients, SGLT1 mRNA levels in tissues from biopsies [68, 100] or nephrectomies [70] were unchanged [70, 100] or raised [68] without any data about protein levels. Regarding SGLT2, its mRNA levels were described as increased [100] or reduced [68, 70] while increased protein content was reported [100]. These very conflicting results can be explained by methodological differences in tissue collection and storage, diabetic status, and possible kidney abnormalities of the control group.

#### 3.2.4. Glucose Effects on Renal Glucose Transporters

In animal models, plasma and luminal glucose concentrations have been shown to stimulate GLUT2 expression [26, 120] and, even, to translocate the transporter from basolateral to BBM side [26]. In canine PT polarized cultures with apical and basolateral cell layers, GLUT2 migrated to the apical side exposed to isolated glucose stimulus [20].

Both SGLTs also seem to be under glucose influence. In cultures of human embryonic kidney (HEK) cells, glucose promoted trafficking of SGLT1 proteins to plasma membrane without changes in the total pool [23] but did not change SGLT1 mRNA levels in PT cultured human kidney-2 (HK2) cells [22]. In addition, glucose stimulated SGLT2 mRNA transcription and amplified SGLT2 protein pool in cultures of human PT cells [22] and promoted its translocation from the intracellular compartment to the membrane in HEK cell cultures [21]. One study, on the other hand, reported a neutral glucose effect on SGLT2 content and/or activity in cultures of human PT cells [121].

#### 3.2.5. Insulin Effect on Renal Glucose Transport

Insulin effects on cells and tissue metabolism result from a highly integrated network of different pathways [122]. IRecs on cell surface, after the insulin binding, phosphorylate the insulin receptor substrate proteins (IRS) that, in turn, activate two main signalling pathways: the phosphatidylinositol 3-kinase (PI3K)/protein kinase B (AKT) pathway, which regulates the majority of insulin metabolic effects, and the Ras-mitogen-activated protein kinase (MAPK) pathway. MAPK participates in the control of cell growth and differentiation through gene expression regulation [122–124]. Insulin itself is the utmost inhibitor of its own signalling [123].

In 1951, Farber at al. demonstrated that insulin decreased Tmax in diabetic patients but under very high insulin plasma levels [48]. However, in a recent trial, physiological insulin levels increased urinary glucose excretion under hyperglycaemic conditions in healthy but not in diabetic volunteers [125]. Both studies separated the insulin effect from glycaemic variation. Thus, emerging questions are as follows: in which way is the higher Tmax of diabetic patients related to insulin resistance, hyperinsulinaemia, or insulin deficiency; and which are the relationships between the insulin signalling and PTs glucose transport proteins activity. That provides a rationale to investigate an insulin effect, isolated or combined to glucose and insulin resistance, on glucose transport proteins, mainly on their function.

Assessing insulin action by itself, a dual temporal insulin effect on glucose uptake was reported in murine PT cultures: raised in the first twenty minutes and returning to the initial rate after thirty minutes [49]. In these cultures, insulin increased GLUT1 mRNA and membrane protein contents but other glucose transporters were not evaluated. Accordingly, regulatory proteins involved in pathways triggered by insulin upregulates the cell surface GLUT1 expression in HEK cell cultures [126]. GLUT1 traffic to the apical membrane in HEK cells has been demonstrated under PI3K/AKT signalling with elevated glucose uptake [127]. The AKT signalling interacts with megalin and the AKT substrate of 160 kDa (AS160), the most downstream insulin signalling step related to insulin-stimulated glucose transport [126, 128]. This signalling reproduces the same insulin-dependent GLUT4 traffic demonstrated in adipocytes [129] and myocytes [130] and could justify GLUT1 raising in HEK and PT cultures exposed to insulin. Concerning renal GLUT2 expression, it was elevated in the presence of insulin resistance, visceral obesity, high triglycerides, and low high-density lipoprotein cholesterol concentrations even under normal glucose levels, in Otsuka Long-Evans Tokushima Fatty (OLETF) rats [25], a T2D model.

About the SGLT system, insulin seems to regulate SGLT1 directly [21] and indirectly [131, 132]. In HEK cell cultures, two hours of insulin exposition inhibited SGLT1 activity [21]. In contrast, the serum and glucocorticoid inducible kinase (SGK1), which is activated by both glucose [133] and insulin [131], stimulated SGLT1 function [132]. The reported findings are very important since the SGLT1 system is virtually fully activated after SGLT2i use or in high glycaemic levels as in diabetes. Besides, SGLT1 is the predominant BBM glucose transporter at PT S3 portion [63, 64], the nephron site with the highest IRec level (46).

Experimental studies indicate SGLT2 activation by insulin. In fact, IRecs seem to be required for maximal SGLT2 expression and SGLT2-mediated glucose reabsorption as evidenced by studies in mice knockout for renal tubule-specific IRecs [134]. Insulin also raised the SGLT2 activity [21, 121] and protein levels [121] independently of glucose concentrations in cultures of human kidney cells. In HEK cells, insulin increased SGLT2 glucose transport by 200 to 300%, probably by stimulating the SGLT2 translocation from an intracellular pool to the S1 and S2 BBM segments [21]. A similar finding was reported using cultured human PT cells where insulin increased SGLT2 content and/or activity in a dose-dependent response [121]. However, in HK2 cells, the activation of the liver X receptor decreases SGLT2 protein and its function. The liver X receptor is a nuclear receptor family that plays a major role in energy metabolism and regulates several membrane transporters. As insulin activates liver X receptor, it could indirectly decrease SGLT2 content [135]. Furthermore, in an Alloxan T1D rat model, insulin reduced SGLT2 mRNA independently of glucose levels [112]. Despite the conflicting data, all these findings open the possibility that the higher SGLT2 levels in diabetic states can be attributed not only to elevated glycaemic concentrations but also to a direct or indirect insulin action. Moreover, insulin resistance perhaps modulates SGLT availability and activity, but this issue was not well evaluated until now.

As insulin resistance is associated with an imbalance of the autonomic system, insulin could indirectly modulate the RTG and Tmax through sympathetic system stimulation. In fact, the reduction of renal sympathetic activity limits SGLT2 excessive transcription in rat models enhancing urinary glucose excretion [22] as well as reducing renal gluconeogenesis in pigs [136].

Organic anion transporters (OAT), proteins situated in the basal membrane of PT cells, contribute to cellular uptake and secretion of multiple molecules to the luminal side, including the SGLT2 inhibitors [137, 138]. The SGLT2i action is related to the SGLTi luminal concentration reached in the S1 and S2 portions and thus depends primarily on the glomerular filtration [139]. However, tubular secretion of SGLT2i [140] mediated by OAT proteins increases its tubular concentration and action [137]. OAT type 3 (OAT3), through its colocalization with SGLT2 but not with SGLT1, enhanced the empagliflozin glycosuric effect [140]. The insulin effect raising [141] and the insulin resistance decreasing [142] renal OAT3 activity on the renal cortex suggest a link between insulin action and pharmacological inhibition of SGLT2. Indeed, a better understanding of insulin effects on tubular glucose transport and its interaction with SGLTi is imperative.

#### 3.2.6. Na^+^K^+^ATPase (NKA)

The ubiquitous NKA protein and its activity have been intensively studied for some decades before our review interval. This transporter is under the influence of many factors, including glucose, catecholamines, C-peptide, insulin, and other hormones [143, 144]. The insulin effect on NKA activity is cell type-specific and depends on the time and intensity of exposition displaying acute and chronic responses [144, 145].

NKA maintains a sodium gradient across the basolateral membrane of PT cells that provides the driving force for the SGLT activity [146]. In this way, changes in NKA activity presumably have an impact on SGLT function and glucose recovery. As insulin influences NKA function and that function directly modulates the SGLT glucose uptake, to evaluate the NKA activity in diabetes can give important information concerning the mechanisms of renal glucose handling regulation by insulin.

Old studies in diabetes models evaluated the NKA activity in the whole kidney and nephron segments, but not in isolated PTs [147], and were inconclusive. Results of recent studies in the whole kidney are still contradictory [148–154] probably because of mixed tissue responses and discrepancies in disease duration and glucose levels.

In the renal cortex of murine STZ models, NKA activity was reported as increased [155–157] or as reduced due to impaired insulin binding to its receptor [158]. In two of those reports with increased NKA activity, insulin treatment reduced it [155, 157]. The duration of disease, i.e., sustained hyperglycaemia or chronic adaptation to it, could have contributed to the differences, as in one study diabetes lasted twice as long as in the other. A specific study on PTs of T2D rats showed a raised NKA activity [159]. In any case, none of these studies investigated the insulin and glucose effects separately.

Although these do not fully represent the real *in vivo* process, cell culture studies evaluating isolated insulin and glucose effects can give a better understanding of the interaction between NKA activity and insulin signalling. Glucose reduced NKA membrane protein and its activity in cultured tubular cells from human nephrectomies [143], and an indirect effect of glucose was demonstrated in HK2 cell cultures where advanced glycation end products reduced NKA activity [160, 161]. An inhibitory glucose effect was also demonstrated in cell cultures of proximal tubule lines from porcine kidneys (LLC-PK1) associated with a downregulation of the surface expression *α*1 subunit, the NKA active site [162, 163]. Thus, glucose seems to be a negative regulator of its own uptake.

Regarding insulin, a short exposition to it (until 30 minutes) raised NKA activity [160, 161, 164], whereas exposition for more than 24 hours reduced NKA activity in rat PT cultures [165]. In the same way, in a culture complex model, insulin exposition raised renal NKA activity in the first 30 minutes, but it returned to the baseline levels after 2 hours and was even lower at 48-hour measurements [166]. This reduction was likewise observed after one hour of insulin exposition in another study [167]. Taken together, these results suggest a dual temporal insulin action on NKA activity. In the NKA low activity second phase, insulin could limit SGLT function by reducing the sodium gradient across the BBM. However, once glucose impacts NKA activity too, the described limiting insulin effect should be evaluated also in the presence of variable and elevated glucose levels, as in diabetes states. Besides, it should be assessed considering a possible renal insulin resistance.

C-peptide is another reported NKA modulator of interest. It increased NKA activity in cultures of human tubular cells from nondiabetic patients [143] and increased NKA alpha subunit mRNA in the renal cortex from STZ rats [168].

### 3.3. Insulin Regulation of Renal Gluconeogenesis

Another important insulin action on PTs is gluconeogenesis inhibition. Liver and renal cortical cells, primarily the PTs [3], are classical tissues that have the enzymatic apparatus necessary to significantly release glucose into the circulation. Hence, PTs contribute to the total endogenous glucose production in fasting and even in postprandial states [47]. Renal glucose release under normal conditions is about 20 to 25% of total systemic glucose production in fasting and 60% in the postprandial state [169].

As kidneys are not able to store significant amounts of glycogen and as glycogenolysis enzymes are lacking, the renal glucose production is provided basically by gluconeogenesis that generates 15–55 g of glucose and kidneys metabolize 25–35 g of glucose per day [47, 170]. Insulin suppresses the renal gluconeogenesis to a lesser extent than it does in the liver probably because of the lower kidney sensitivity to this insulin effect. However, such a difference could be the result of lower insulin delivery to the renal tissue. Furthermore, glucagon has little to no effect on renal gluconeogenesis [170–172]; hence, catecholamines are the major counter regulator of insulin-induced inhibition of gluconeogenesis in the kidneys [170, 172].

Renal gluconeogenesis is enhanced in STZ rats [4, 14, 46, 67, 110, 173], in murine model knockout for IRecs [174] or IRS1 plus IRS2 [4], in a mix model of high-fat diet plus STZ [175], and in T2D murine models [176, 177] demonstrating the essential insulin role.

Reabsorbed glucose from tubular filtrate [4, 178] and insulin [4] seems to have a complementary inhibitory effect on renal gluconeogenesis. In fact, the higher postprandial insulin levels reduce PT gluconeogenic enzyme transcription in wild mice [4] and rabbits [179], and gluconeogenic gene expression was reduced by the glucose counterregulatory effect in insulin-resistant and insulinopenic models [4]. In addition, SGLT1 plus SGLT2 inhibition by phlorizin restored gluconeogenic activity in these models [4] and isolated SGLT2 inhibition in normal mice activated renal gluconeogenic gene expression [178]. Therefore, the reduction of glucose flux across PT cells stimulates gluconeogenesis. Moreover, in HK2 cell cultures, insulin and glucose inhibit gluconeogenic enzymes across distinct pathways [4].

In accordance, PT cells from human nephrectomies [176] and HK2 cell cultures [4] exposed to insulin undergo gluconeogenesis reduction. However, a high gluconeogenic enzyme content in human renal biopsies from T2D was reported [46] which could be interpreted as an impairment of insulin action on kidneys, maybe a kidney-specific insulin resistance. The intracellular glucose generated from high-intensity gluconeogenesis might impact the glucose transport through modifications of SGLT2 transcription or its pool mobilization, as described for the extracellular glucose stimulus in PT cells of diabetes models. That could mean an additional indirect insulin regulation of glucose transport, in this case, through gluconeogenesis.

### 3.4. Renal Insulin Resistance

Despite the higher Tmax for glucose in diabetic patients compared to healthy subjects, it is not clear if renal insulin resistance could impact glucose transport. Even the concept of renal insulin resistance is still debatable. Insulin resistance, in general, is characterized by an attenuation of its triggered biologic processes inducing metabolic impairment [123, 180, 181], and the insulin resistance phenotype is variable among organs and even among tissues from the same organ. For example, the liver has selective insulin resistance, and metabolic pathways diverge according to specific spatial zonation near or distal to the portal space [123]. The same may be possible in different renal segments according to the presence and density of IRecs and insulin availability considering the hormone filtration, extraction, and degradation.

The variability of protein isoforms of the insulin signalling cascade (IRecs, IRS, PI3K, and AKT) [122] and of diabetes phenotypes, mainly in T2D [182, 183], is partially due to genetic variations [184–186] and may be related to specific tissue resistance differences. In addition, insulin signalling determines several phenotypic characteristics regarding cell size and proliferation in PTs [187]. Therefore, another question is if the insulin action on PT glucose transport is impaired in insulin resistance.

The two IRec isoforms differ in affinity to insulin binding and metabolic effects [188, 189]. In humans, IRec type B, available mainly in insulin-sensitive organs (skeletal muscle, liver, and adipose tissue) [188, 190], is abundant in kidneys too [190]. In rat models, insulin binding [50] and IRecs are present along the whole nephron with the highest levels at PTs, especially in the outer medullary S3 portion [46]. The distal convoluted tubule is another nephron segment where insulin binding is high [50] and where insulin stimulates sodium reabsorption [180, 191, 192]. At PTs, insulin stimulates sodium uptake also through Na^+^H^+^ exchanger type 3 (NHE3) [180].

The differences in IRec density and of insulin concentration along the nephron indicate a specific site and variable hormone action. Some findings in animal cell cultures demonstrated variations of nephron or PT IRec densities. In PTs of normal rats, IRecs are localized in the basolateral membrane where it may sense insulin from capillaries while IRec on the apical membrane is involved in insulin reabsorption [44, 46]. IRecs accumulate into the cytoplasm during fasting and in the two membranes after refeeding consequent to both insulin and glucose oscillations [46]. Insulin decreases its own receptors in murine PT cultured cells [165]. Reduced IRec protein expression in all nephron segments in either insulin-resistant [193] or insulinopenic rats [46] has been described. The latter had a stronger reduction in the renal cortex and distal tubules [46]. The increase of membrane IRecs after feeding was also lost in diabetes models [46]. In humans, IRec protein expression was also significantly reduced in renal biopsies from T2D patients with a pronounced downregulation observed in PTs and slightly in distal tubule cells [46] again suggesting reduced insulin action on PTs.

Impairment of another step of the insulin signalling cascade in PTs has been described. After the IRS phosphorylation triggered by the insulin binding, the IRS tyrosine residues serve as anchoring sites for regulatory subunits of PI3K at the cell membrane cytoplasmic side [194]. The IRS1 and IRS2 isoforms, widely expressed in human tissues, have distinct physiological roles *in vivo* [33] and are frequently decreased in insulin-resistant states [124]. Hyperinsulinaemia induces IRS1 and IRS2 protein degradation [195] across different pathways [124], according to the target organ where the insulin resistance takes place. In PTs of insulin-resistant murine models, the stimulatory effect of insulin via IRS1 is impaired in contrast to a preserved IRS2 insulin signalling [180]. IRS2 has a role in PT sodium transport not related to the SGLT system [121, 196]. On the other hand, IRS1 impaired signalling may be associated with a lesser inhibition of renal gluconeogenesis [46, 47, 197]. While IRS1 expression and phosphorylation are normal [198] or reduced [199], IRS2 has normal levels in diabetes models [27, 191]. IRS2 expression is preserved in the renal cortex of insulin-resistant patients [191] or even enhanced in tubules of patients with diabetic nephropathy [200]. These findings corroborate the renal insulin resistance hypothesis as well as a site-specific and selective resistance. It is reasonable that a PT insulin resistance, beyond being related to an impaired gluconeogenesis regulation, could impact renal glucose transport and thus hypothetically contribute to the higher Tmax found in diabetes.

Other corroborating evidences are the increased inflammatory markers (NF-*κ*B, TNF*α*, IL-6, and IL-10) reported in cortical tissues of murine diabetes models [201–203], HK2 cell cultures under high glucose environment (NF-*κ*B) [204], and cortical portions of T2D patients (NF-*κ*B) [202]. These elevated markers were associated with disrupted insulin signalling characterized by high FOXO1 and reduced AKT [202], PPAR*γ*, and ISRS1 [201, 203] but maintained ISR2 levels [201]. Increased renal gluconeogenesis [202], as expected, and reduced GLUT2 [203] were also associated with enhanced inflammatory markers.

## 4. Summary of Evidence and Discussion

The review objective was to describe and summarize the literature data about the insulin effect on renal glucose transport. We aimed to construct a sequence of evidence to facilitate the reader access to the current understanding of insulin action on renal proximal tubules, the nephron site responsible for the glucose uptake from glomerular filtrate, and where renal gluconeogenesis takes place. In the following paragraphs, the main findings are summarized.

Kidneys, mainly PTs, play a significant role in insulin metabolism. Insulin upregulates its own PT uptake and degradation [41], thus changing insulin availability in the whole body and specific renal sites [54, 55].

Regarding glucose transporters in diabetes, T1D models showed increased GLUT1 protein availability and mRNA expression in the whole kidney and higher cortical GLUT1 mRNA expression. These changes can be transitory and site-specific. Results concerning GLUT2 are controversial. SGLT1 studies agreed only in the upregulation of its mRNA expression in T2D models while protein and mRNA SGLT2 contents in both T1D and T2D models are frequently reported as increased (Table 1). Elevated SGLT2 levels could explain the higher glucose uptake capacity of diabetic patients. Human studies, however, are scarce and contradictory with few studies demonstrating raised SGLT2 protein availability in diabetic patients.

Insulin alone [21, 121] or with glucose [24, 25] can modulate availability and/or function of PT glucose transporters beyond changing renal gluconeogenesis [4, 178]. The insulin effect in murine PT cell cultures seems to increase GLUT1 content and trafficking [49, 126]. Insulin resistance, on the other hand, is associated with increased GLUT2 in animal models [25] while insulin replacement reduces this transporter availability [24]. However, glucose level variations may have confused the results in these models. While glucose has promoted SGLT1 trafficking [23], insulin seems to directly inhibit the SGLT1 activity in renal human cell cultures [21] but could activate it indirectly [131]. Furthermore, glucose seems to amplify membrane SGLT2 protein availability in these cultures [22]. It was reported that insulin raises SGLT2 protein availability and activity independently of glucose and additionally regulates SGLT2i bioavailability [140–142]. Differences in IRec density along the nephron [46] and in the type of IRS expressed in diverse tubule segments, or the same segment but under distinct insulin sensitivity [27, 191, 199–201, 203], point to a renal site-specific selective insulin action and, possibly, to a spatial selective insulin resistance.

Insulin action on the sympathetic system can, indirectly, modulate SGLT2, hence changing glucose handling [22]. In addition, renal gluconeogenesis is enhanced in diabetes [4, 14, 46, 67, 110, 173–177] and is inhibited by insulin [4, 179], which could influence glucose reabsorption through SGLT2 [21, 22].

NKA activity might impact SGLTs by providing the driving force for their activity [146]. In murine models of diabetes, changes in NKA function are probably due to high glycaemic levels [155–157, 159] and impaired insulin signalling [158]. Nevertheless, the results' heterogeneity does not allow to clearly define the insulin effect on NKA. In complex models of animal PT cultures, NKA activity increased after short exposition to insulin but decreased under sustained stimulus [160, 161, 164–167]. In human tubular cell cultures, glucose inhibited while C-peptide stimulated NKA activity [143, 168].

**Figure 2:**
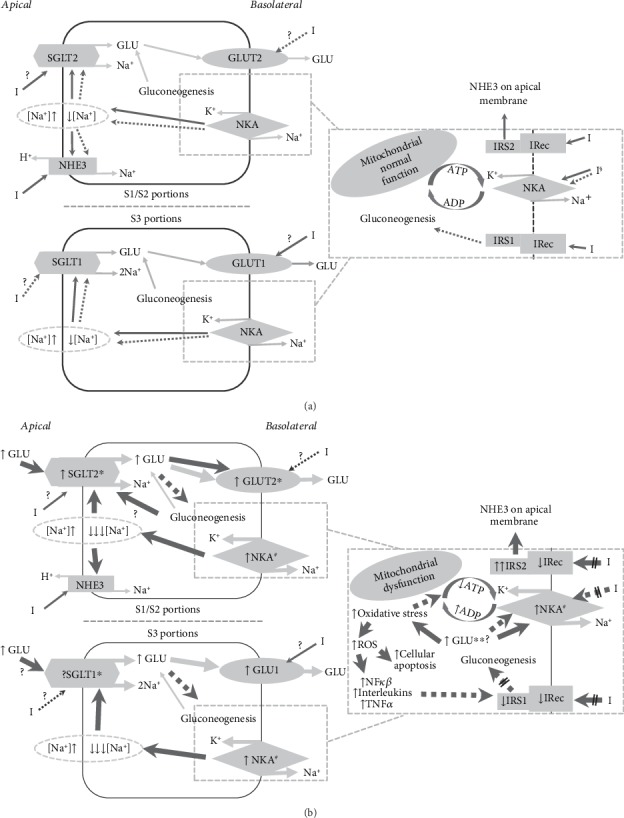
(a) Insulin effect on renal proximal tubule cells. (b) Diabetes, hyperinsulinaemia, and insulin resistance impact on renal proximal tubule cells. Grey arrows = flux; black continuous arrows = stimulatory effect; black interrupted arrows = inhibitory effect; thicker arrows = increased activity. I: insulin; GLU: glucose; ROS: reactive oxygen species; NHE3: Na^+^H^+^ exchanger type 3; NKA: Na^+^K^+^ATPase; IRecs: insulin receptors; IRS: insulin receptor substrate proteins; NF-*κ*B: nuclear factor kappa-light-chain-enhancer of activated B cells; TNF*α*: tumor necrosis factor alpha; IL-6 and IL-10: interleukins. ^?^Scanty or conflicting data; ^//^reduced effect; ∗enhanced in animal models but conflicting human data; ∗∗enhanced in murine models but reduced in cultures; ^§^temporal dual action according to exposition (short time = stimulatory and sustained = inhibitory); ^#^total NKA function increased despite inhibitory GLU effect and mitochondrial dysfunction.

All of the above findings are summarized in Figures 2(a) and 2(b).

Therefore, the elevated Tmax of diabetic patients [16, 18, 19, 48, 118] yet so far not completely known is possibly associated with an upregulation of glucose protein transporters and may be related to insulin in many ways. Human studies with reproducible and comparable methodology are needed to understand the real impact of insulin on glucose transport in healthy and diabetic subjects, independently of glucose influence.

Our review has limitations. It is circumscribed to publications in the last 10 years. The literature search using specific terms and the limitation to publications in English may have missed some papers related to our aim. Other difficulties are related to the issue itself. In fact, most studies did not have the insulin action on glucose transport as their first objective. Results are not always comparable taking into account differences among species [102, 189, 205] and study models. In human studies, one limitation is the inclusion of subjects with other kidney diseases as the control group rather than just healthy ones. Moreover, frequently, insulin and glucose effects were not evaluated separately. Cell culture models are able to isolate these effects although they do not consider the microenvironment of the whole organ, possibly influencing transcriptional regulators of genes involved in glucose utilization [49, 206], and do not consider the hormonal [49, 207–209] and neural [22, 209] crosstalking among organs. It is still important to take into account that mRNA or protein measurements do not necessarily reflect their dynamic function. At the same protein content, its function can be enhanced or diminished by modification of serum lipids and fluidity in the cytoplasmic membrane [144], by transporter conformational changes [5] or by subcellular spatial arrangement [67, 210]. Furthermore, protein interactions in the cytoplasmic membrane side, as described for SGLT2 and its anchoring protein [211], can be related to variation in glucose transporter function without any change in the protein content [210, 212].

In conclusion, the upregulation of renal glucose transporters, mainly SGLT2, associated with sustained hyperglycaemia, or to a disrupted renal insulin signalling, can be related to the increased maximum renal glucose reabsorptive capacity observed in diabetes. The several effects of insulin on distinct kidney sites can modify glucose transport directly, through changes of glucose transporter availability and function, or indirectly through Na^+^K^+^ATPase activity modulation. Thus, there is evidence of insulin effect not only on renal gluconeogenesis but also on renal glucose transport. However, until now the scarcity and the heterogeneity of the studies limit an accurate proposal of the implicated mechanisms.

## Figures and Tables

**Table 1:** Glucose transporter protein and mRNA availability in T1D and T2D murine models.

	Results	Protein	mRNA
GLUT1∗			
T1D	Increased	[79–82] (STZ^WK^); [86, 87] (STZ^C^)	[81–85] (STZ^WK^); [86, 88, 89] (STZ^C^)
	Similar	[93] (STZ^WK^); [67, 91] (STZ^C^)	[93] (STZ^WK^)
	Reduced		[92] (STZ^WK^)
T2D	Increased		[85] (db/db^WK^)
	Similar	[90] (GK^C^); [90] (JK^C^); [90] (HFD^C^)	[22] (OLETF^C^)
	Reduced		[92] (GK^WK^)
GLUT2			
T1D	Increased	[26, 79, 87, 90, 96] (STZ); [99] (Alloxan)	[24, 83] (STZ); [84] (Akita); [97] (Alloxan)
	Similar	[91] (STZ)	[85, 91, 92, 93, 98] (STZ); [96] (Akita)
	Reduced	[95] (Alloxan)	[95] (Alloxan)
T2D	Increased	[25] (MG); [94] (Zucker)	[22] (OLETF); [100] (db/db^§^)
	Similar	[90] (GK); [90] (JK); [90] (HFD)	[85] (db/db); [92] (GK); [98] (HFD)
SGLT1			
T1D	Increased	[80] (STZ)	[83] (STZ); [84] (Akita)
	Similar		[93, 98] (STZ)
	Reduced	[84] (Akita); [103] (STZ)	[85, 92] (STZ)
T2D	Increased	[90] (GK); [90] (JF); [103] (db/db); [105] (HFD^#^)	[104] (OB/OB); [100] (db/db^§^); [22] (OLETF); [106] (Zucker); [92] (GK)
	Similar	[100] (db/db^§^)	[98] (HFD)
SGLT2			
T1D	Increased	[32, 67, 107, 108, 109], [110]^a^ (STZ); [84, 85] (Akita); [99] (Alloxan)	[32, 83, 107, 108, 109] (STZ); [112] (Alloxan)
	Similar	[93] (STZ)	[93] (STZ)
	Reduced	[85]^b^, [113]^a,c^ (STZ)	[85]^b^, [98] (STZ)
T2D	Increased	[90] (JF); [111] (db/db); [94] (Zucker)	[85] (db/db); [100] (db/db^§^); [22] (OLETF); [106] (Zucker); [104] (OB/OB)
	Similar	[100] (db/db^§^); [105] (HFD^#^)	
	Reduced		[98] (HFD)

Results were compared to the corresponding controls; numbers are references; the study model is inside the parentheses. ∗Results for GLUT1 were specified for whole kidney (WK) or cortex (C) due to the different availability of GLUT1 in distinct nephron sites, while GLUT2, SGLT1, and SGLT2 are available only at proximal tubules level. ^a^Short-duration diabetes. ^b^Initially reduced followed by a partial recovery but maintaining lower levels. ^c^Protein activity was also reduced. STZ: streptozotocin model; db/db: leptin receptor mutation model; GK: Goto–Kakizaki diabetic rats; HFD: high-fat diet; OLETF: Otsuka Long-Evans Tokushima Fatty rats; MG: monosodium glutamate treatment. ^§^Mix model with insulinopenic and insulin-resistant rats. ^#^Insulin resistance without changes in glycaemic levels compared to controls.

## Abbreviations

AKT: Protein kinase B
BBM: Brush border membrane
GLUT: Facilitative glucose transporter
HEK: Human embryonic kidney
HK2: Human kidney-2
IRecs: Insulin receptors
IRS: Insulin receptor substrate proteins
NKA: Na^+^K^+^ATPase
PI3K: Phosphatidylinositol 3-kinase
PTs: Proximal tubules
S1 PTs: Proximal segment
S2 PTs: Intermediary segment
S3 PTs: Distal segment
SGLT: Sodium-glucose linked transporter
SGLT2i: SGLT2 inhibitors
STZ: Streptozotocin
RTG: Renal threshold for glucose
T1D: Type 1 diabetes
T2D: Type 2 diabetes
Tmax: Maximum renal glucose reabsorptive capacity.
